# Assessing the implementation of user-centred design standards on assistive technology for persons with visual impairments: a systematic review

**DOI:** 10.3389/fresc.2023.1238158

**Published:** 2023-09-06

**Authors:** Luisa Maria Ortiz-Escobar, Mario Andres Chavarria, Klaus Schönenberger, Samia Hurst, Michael Ashley Stein, Anthony Mugeere, Minerva Rivas Velarde

**Affiliations:** ^1^ Institute of Ethics, History, and Humanities, University of Geneva, Geneva, Switzerland; ^2^ Vicerrectoría de Investigaciones, Universidad Autónoma de Occidente, Cali, Colombia; ^3^ EssentialTech Centre, EPFL, Lausanne, Switzerland; ^4^ Harvard Law School Project on Disability, Harvard University, Cambridge, MA, United States; ^5^ Harvard Law School, Harvard University, Cambridge, MA, United States; ^6^ Faculty of Law Centre for Human Rights, University of Pretoria, Pretoria, South Africa; ^7^ Department of Sociology and Anthropology, Makerere University, Kampala, Uganda

**Keywords:** visual disability, visually impaired, assistive technology (AT), user–centered design, human centred design, innovation, low and middle income countries (LMIC), technology design

## Abstract

Despite scientific and technological advances in the field of assistive technology (AT) for people with visual impairment (VI), technological designs are frequently based on a poor understanding of the physical and social context of use, resulting in devices that are less than optimal for their intended beneficiaries. To resolve this situation, user-centred approaches in the development process of AT have been widely adopted in recent years. However, there is a lack of systematization on the application of this approach. This systematic review registered in PROSPERO (CRD42022307466), assesses the application of the ISO 9241-210 human-centred design principles in allegedly “user-centred designed” AT developments for persons with VI (see Supplementary PROSPERO Protocol). The results point to a wide variation of the depth of understanding of user needs, a poor characterization of the application of the User Centred Design (UCD) approach in the initial design phases or in the early prototyping, and a vague description of user feedback and device iteration. Among the principles set out in ISO 9241-210, the application of 5.6: “the design team includes multidisciplinary skills and perspectives” is the one for which the least evidence is found. The results show there is not enough evidence to fully assess the impact of UCD in (1) promoting innovation regarding AT products and practices, and (2) Judging if AT produced following such standards is leading to better user access, wellbeing outcomes and satisfaction. To address this gap it is necessary to, first, generate better implementation of UCD in AT development and second, to strengthen evidence regarding the implementation and outcomes of using UCD for AT. To better engage with the realities of persons with VI, we propose capacity building across development teams regarding UCD, its principles and components; better planning for UCD implementation; and cross-fertilization across engineering disciplines and social and clinical science.

**Systematic Review Registration:**

https://www.crd.york.ac.uk/PROSPERO/display_record.php?RecordID=307466 PROSPERO (CRD42022307466).

## Introduction

User-Centred Design (UCD) has gained a stronger presence in Assistive Technology (AT) development over the last decade (1). This approach promotes the involvement of end users in all stages of the design process, elicitation and understanding of their needs, and characterization of social contexts as the basis for an iterative design process (2, 3). Therefore, UCD adoption is believed to lead to better products (4). However, there is limited evidence regarding the implementation of these approaches or if their results are having the intended impact across their target populations, particularly regarding AT (5, 6). This study aims to assess the application of ISO 9241-210 human-centred design principles in the allegedly “user-centred designed” assistive technology developments for persons with Visual Impairments (VI).

The Global Report on Assistive Technology (GREAT) states that children and adults with disabilities lack access to AT, particularly in low-and-middle-income Countries (LMICs) where access was reported to be as low as 3% (7). The current lack of access to AT reflects not only an economic gap but a severe malfunction of social provision and coverage schemes as well as in AT design and development (8). Nevertheless, UCD and international standards' adoption can help to alleviate these shortfalls by guiding the development of better and more efficient AT solutions responding to the users' priorities. Disability is very diverse and persons with different impairments, namely sensorial, physical or cognitive or multiples, benefit from different technological solutions; we need to learn more about similarities, as well as differences. Therefore, in this paper, the focus is on AT for persons with VI. Worldwide, there are approximately 39 million people with severe VI or blindness (9). Although not all-disabling loss of sight can be addressed by AT, for persons who are blind (visual acuity worse than 3/60) some tools such as walking canes, screen readers, or braille embossers, amongst others, are of great help. The Global report on disability calls for action and standard setting in a variety of AT related fields, particularly regarding access (7). Thus, investigating how internationally adopted standards are implemented for technology design is relevant to close the AT gap. The upcoming section explores relevant international standards for the production of AT.

### AT and international standards

International standards play a key role in the development, production and distribution of technology (10). The existence of clear, accessible, and commonly accepted International Standards is vital for the manufacture of products that can be globally implemented and commercialized. Standardization enhances product quality, safety, and reliability, it also allows for higher interoperability and compatibility in different contexts and reduces maintenance complexity and costs (11). There are different studies on the positive effect of international standardization on trade, industry and management (10, 12), including evidence of the reduction of barriers to the export and acceptance of products between different global regions, including products exported from LMICs to high income Countries (13). Given the current AT access gap and lack of evidence on how available technology responds to the needs of persons with VI in LMIC it is relevant to look at how and whether the adoption of these standards can lead to better and more efficient AT. Furthermore, infrastructure for AT production and a well-defined value chain have an impact on AT access, nonetheless, this is outside the scope of this paper.

### The standard of user-centred design

UCD is recognized by the International Organization for Standardization (ISO) in their standard ISO 9241-210, where it is described as an “approach to system design and development that aims to make interactive systems more usable by focusing on system use and applying human factors/ergonomics and usability knowledge and techniques” (3). The standard presents a framework giving examples of activities that can be developed when adopting the approach. Furthermore, it clarifies that UCD is complementary to existing design methodologies, for example regarding usability (14) and Measurement of quality in use ISO/IEC 25022, amongst others. UCD is guided by the following 6 principles: (I) the design is based upon an explicit understanding of users, tasks and environments; (II) users are involved throughout design and development; (III) the design is driven and refined by user-centred evaluation; (IV) the process is iterative; (V) the design addresses the whole user experience; and (VI) the design team includes multidisciplinary skills and perspectives.

There is very narrow empirical evidence on the impact of standards on innovation, particularly regarding AT (15). However, forthcoming empirical literature shows a positive influence of standards on the diffusion of technical knowledge and their contribution to macroeconomic growth. For example, a set of studies performed within different countries showed that the contribution of standards to the growth rate in each of the evaluated countries was equivalent to “0.9% in Germany, 0.8% in France and Australia, 0.3% in the UK and 0.2% in Canada” (12). Another set of studies, performed by the ISO in several companies from different sectors in ten countries, showed an overall increase between 0.5% to 4% in the companies’ annual sales revenues provided by the implementation of international standards (13, 16, 17).

The adoption of a user-centred design approach in the development process of AT has increased in recent years. This systematic review assesses the application of the ISO 9241-210 Human-centred design principles in the “user-centred designed” AT developments for persons with VI. The goal is to better understand how systematically the approach has been applied in the design and development of AT.

## Method

The present review followed the Prisma guidelines for systematic reviews seeking to answer the next question (Supplementary PRISMA Checklist) (18).

*Does the existing literature provide sufficient evidence on how developments of user-centred designed assistive technology for persons with visual impairments apply the human-centred design principles of the ISO 9241-210?*

The multidisciplinary databases Science Direct, Scopus, PubMed Central and Web of Science, were defined as primary sources. The electronic searches were performed in January 2022 and updated on June 2022. The keywords visual impairments (blindness and low vision), user-centred design, and assistive technology were used as search terms. The search period was established between January 2012 and March 2022. Considering that standards take time to become known and applied, a gap of two years was left between the publication of ISO 9241-210 (2010) and the start date of the search (2012). In any case, the application of the previous standard (ISO 13407) was considered during the revision. The complete Web of Science search strategy, was adapted for the other databases:

Search string: [(“visual+ impair+” OR “visual+  disab+” OR blind OR “low vision”) AND (“user-centred design” OR “human-centred design” OR “ISO 9241-210” NOT “universal design”) AND (assistive technology)]

### Eligibility criteria

#### Inclusion criteria

• Topic of study: papers are describing the design and/or development process of user centred designed assistive technology for visually impaired persons.
• Type of scientific material to analyse: Peer-reviewed journals: Quantitative, qualitative, and mixed methods empirical studies. Except for systematic reviews and meta analyses, letters and editorials.
• Studies available in English/Spanish/Portuguese/French.
• Full text available.
• Full conference papers.

#### Exclusion criteria

• Articles that are not exclusively addressed to persons with VI.
• Articles describing assistive technology design or developments addressed for persons with VI without any consideration to the UCD approach.

### Study selection

All search results were imported into an EndNote database. Duplicates were removed. Abstracts and titles that were noticeably unrelated to the review topic were dismissed. Two researchers independently screened the titles and abstracts against the eligibility criteria and selected those that met the inclusion criteria. Full-text reports were retrieved and again assessed for final eligibility. Reasons for excluding full-text reports were documented. The selected studies were analysed with a standardised data extraction form. Inter-rater agreement was 87.12%, Disagreements during the selection process were discussed in a consensus meeting with a third reviewer who helped to solve the discrepancies.

### Data extraction

To meet the aim of the study, all data related to the application of each of the six principles of ISO 9241-210 was extracted and grouped under each of the principles for further analysis.

The following data on the study characteristics were also extracted for contextual purposes:

• Data about the publication (authors, title of the article and the journal), aims, methods, design approaches (usability testing, workshops, interviews, focus groups, think-aloud, observation, including others.), frameworks, and studies design.
• Data about the designs or developments: areas covered (according to the ISO 9999:2016).
• Data about participants: sample size, socio-demographic characteristics, inclusion and exclusion criteria, type of impairments (low vision or blindness).
• Setting: country.

### Data quality assessment

The methodological quality of the selected studies was assessed using the Mixed Methods Appraisal Tool (MMAT) (19). This tool was selected for its applicability in evaluating qualitative, quantitative, and mixed methods empirical studies. Studies were assessed over five components which vary according to the nature of the study. For the qualitative studies, the following criteria were assessed: The appropriateness of the qualitative approach (1), the data collection methods (2), and the data analysis (3) to answer the research question. In addition, that the resultś interpretation was supported by the data (4), and the coherence between all these parts of the study (5). Regarding the quantitative studies, the assessment addressed (1) the relevance of the sampling strategy according the research question. (2) The representativeness of the sample. (3) The validity, reliability and coherence of the measurements. (4) The minimization of the risk of nonresponse bias, and (5) the appropriateness of the statistical analysis. The items evaluated for mixed methods studies, were: (1) the pertinence for applying a mixed methods design to answer the research question. (2) The integration of the quantitative and qualitative components of the study. (3) The general interpretations resulting from consolidation of qualitative and quantitative results. (4) The presentation and explanation of dissonances between the components findings. (5) The quality criteria of each of the components of the study. The instrument encourages not to estimate an overall score to rate the quality of each study, but to analyse and discuss each criterion. It was implemented independently by two researchers to perform, and disagreements were resolved through discussion with a third researcher. Following the indications of the MMAT, studies were not excluded based on the methodological quality. Instead, the results of the evaluation are addressed in the discussion and conclusions. Two of the authors jointly (MRV and LMO) assessed the risk of bias and quality of the articles. Disagreements were resolved by consulting (MACh) to achieve consensus.

### Data analysis and synthesis

All search results and their respective reasons for inclusion or exclusion were documented through The PRISMA flowchart. The qualitative evidence was analysed and synthesised using thematic synthesis approach guided by the Human-centred design approach described in the ISO 9241-210. The analysis and synthesis was carried out by two researchers. A third researcher assessed Inter rater reliability.

### Protocol and registration

The protocol describing this systematic review methodology was previously registered in PROSPERO (CRD42022307466).

## Results

**Figure 1:**
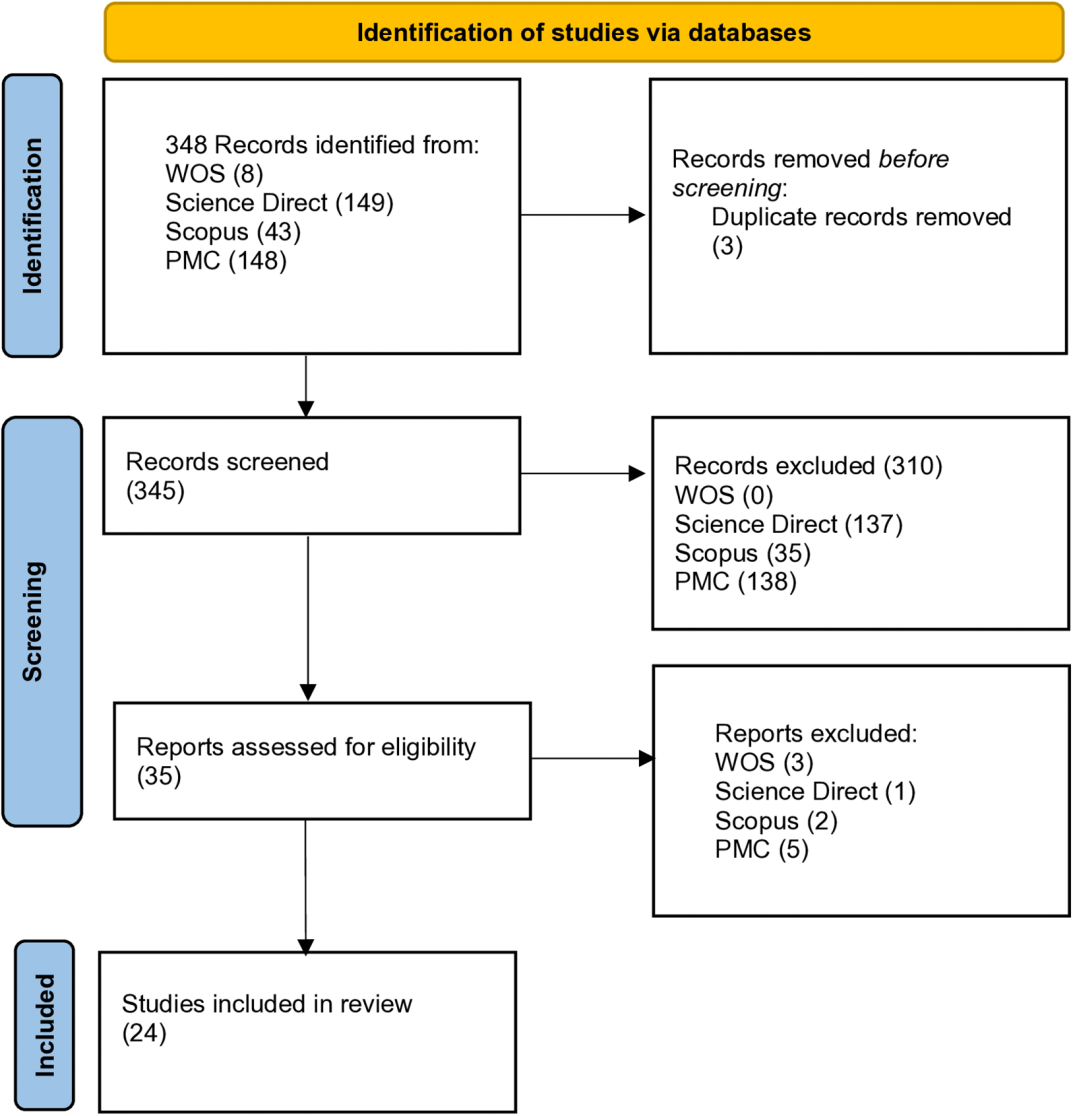
Prisma flow diagram.

The search in databases retrieved 348 references, After removing three papers that were duplicated, 310 papers were discarded through the abstract screening stage. The remaining 35 were subjected to a full-text review. Among them, four literature reviews and seven empirical studies were discarded for not meeting the inclusion criteria (see Figure 1).

### Overview of included studies

#### Themes covered in the literature and geo-representation

The results retrieved contributions from 16 countries. Regarding countries' distribution by income category, it should be noted that 87.5% (24) of the papers included are from high-income countries, four from middle-income, and none from low-income countries (Table 1). AT designs or developments covered the following areas (according to the ISO 9999:2016) (20) https://sciwheel.com/work/citation?ids=14235244&pre=&suf=&sa=0: activities and participation relating to personal mobility and transportation (ten papers); communication and information management (five papers); education and training in skills (six papers); work activities and participation in employment (one paper); and for assistive products for self-care activities and participation in self-care (two papers).

**Table 1:** Number of studies per country.

Country	No of studies	Classification by income level
Austria	1	HIC
Belgium	1	HIC
Brazil	1	MIC
Chile	1	HIC
France	2	HIC
Germany	1	HIC
Greece	1	HIC
Italy	2	HIC
Malaysia	1	MIC
Portugal	1	HIC
Saudi Arabia	1	HIC
Spain	1	HIC
Taiwan	1	HIC
Thailand	1	MIC
UK	1	HIC
USA	7	HIC

The countries of origin of the studies included in the review and their classification by income level according to World Bank data (2022), are presented.

HIC, High income country; MIC, Middle income country.

Most of the papers (21/24) explicitly declare using/implementing the UCD framework of reference. However, the implemented framework, namely the ISO 9241-210 (or its previous version ISO 13407) was referenced in only five of the reviewed articles (21–25). Excluding the aforementioned studies, only three papers (22, 26, 27), cited a reference, other than ISO 9241-210, for UCD, specifically Cheverst et al. (28) and Nielsen (29).

Further characteristics of the studies included in the review are summarised in Table 2. These data contextualise the results of both the quality assessment of the studies (e.g., aims of the studies, data on population samples, data collection instruments, etc.) and the application of the principles of ISO 9241-210 (e.g., characteristics of participants and their involvement in the studies, among others), which are presented below.

**Table 2:** Characteristics of the included studies.

Authors	Article title	Publication year	Aim	Sample	Data collection tools, techniques
Conradie et al. (21)	Participation is Blind: Involving Low Vision Lead Users in Product Development	2015	To present how the Lead User approach was applied, focusing on the tools and techniques used.	For interviews: 6 Visually impaired persons. For focus groups: FG1: nine participants; FG2: 12 participants. Lead users: one participant	Qualitative interviews, focus groups, Bodystorming technique. Knigth`s Tool.
Façanha et al. (22)	Design and evaluation of mobile sensing technologies for identifying medicines by people with visual disabilities	2019	To assist people with visual impairments to identify their medicine by using mobile sensing technologies.	Two blind adults participated in the design phase; and ten in the usability test.	Usability evaluation instruments (SUS and SUBC), accompanied by a brief interview about the users’ impressions
Ntakolia et al. (23)	User-centered system design for assisted navigation of visually impaired individuals in outdoor cultural environments	2020	To develop a human–computer interactive system addressed to guide VIIs in outdoor cultural environments.	For the focus group stakeholders (no determined number of participants). For the questionnaires: 51 VI and 77 non VI participants. For the evaluation of the prototype: 10 blinded users.	Observations in the outdoor environment, Focusgroups-interviews, questionnaires, prototype usability test
Mattheiss et al. (24)	User-centred design with visually impaired pupils: A case study of a game editor for orientation and mobility training	2016	This aim raises open research questions on how to design an accessible O&M training game editor.	25 teenagers and young adults from several classes of a school for VI pupils in Vienna, Austria, with business and polytechnical focus.	Semi-structured expert interview Workshop about brainstorming methods, digital survey, Focus group, Behaviour observation and contextual, exploratory interview in game playing, Self-experience in O&M training
Sánchez (25)	Development of Navigation Skills Through Audio Haptic Videogaming in Learners Who are Blind	2012	To investigate whether the use of audio and haptic-based videogame has an impact on the development of O&M skills in school-age blind learners.	For the usability evaluation: 10 blind learners (ages from 9 to 15 years). None of these participants have any additional, associated disabilities other than VI. Cognitive impact test: 7 blind learners (10 and 15 years old).	Interaction data collected from independent onsite user evaluations, using observation together with a think-aloud protocol, field notes, semi-structured interview and a specialized checklist.
Nimmolrat et al. (26)	Pharmaceutical mobile application for visually-impaired people in Thailand: development and implementation	2021	To analyse, design and implement a mobile pharmaceutical application, which enables users to manage their medication	Sixty volunteers from the Vision Disability Association in northern Thailand. The inclusion criteria and sampling methods are presented.	Interviews for the Identification of users’ problems and needs. Feedback and ranking based in a usability questionnaire.
Yeh and Yang (27)	Assisting the visually impaired to deal with telephone interview jobs using information and commutation technology	2014	To develop a new ICT assisted blind telephone interview (ICT-ABTI) system to increase the working performance of the visually impaired.	Seven visually impaired people. All of the participants graduated from university and had enough experience in using computers. The ethnic backgrounds are presented. Participant's ages ranged from 24 to 39 years of age.	Interviews, ABAB design to execute the tests, in which A represented the baseline phase (without ICT-ABTI system) and B represented the intervention phase (with ICT-ABTI system).
Mascetti et al. (30)	Sonification of guidance data during roadcrossing for people with visual impairments or blindness	2015	Two original auditory guiding modes based on data sonification are presented and compared with a guiding mode based on speech messages	Three sets of empirical evaluations were conducted: quantitative evaluation with 11 blindfolded sighted test subjects, a qualitative evaluation with 12 blind test subjects and, finally, a quantitative and qualitative evaluation conducted with 3 test subjects with VIB.	"Informal evaluations, preliminary evaluations”. Experimental tests: quantitative variables related with time responses to the audio cues. Qualitative tests: The questionnaire is organized in two sets of Likert-scale items (derived from the SUS and from IBM Computer Usability Satisfaction Questionnaire) and an additional set of open questions.
Najjar et al. (31)	Dynamic indoor path planning for the visually impaired	2022	To facilitate the navigation process for the VI, through developing a useful indoor navigation mobile application that can safely lead them to the desired destination.	17 participants in total, 10 non-VI volunteers, and 7 VI participants.	Usability test in which effectiveness was measured through accuracy to detect deviation. Efficiency through the time to complete a task. And comfort and acceptability, thorugh The System Usability Scale.
Connors et al. (32)	Action video game play and transfer of navigation and spatial cognition skills in adolescents who are blind	2014	We have developed a novel approach to train navigation and spatial cognition skills in adolescents who are blind.	Seven early blind adolescents aged between 16 and 17 years (3 males) familiar with the use of a computer key board interface	Number of correct paths executed (expressed as mean percentage correct). Conversations,interviews,and usability evaluations, to gather input from potential end-users as part of the user-centred design employed, are mentioned but not described in the paper.
Aziz et al. (33)	User Experience of Interactive Assistive Courseware for Low Vision Learners (AC4LV): Initial Round	2017	To investigate user experience of AC4LV in terms of information accessibility, navigationability, and pleasurability.	Eight subjects with the average age 9 to 12 were involved.	Subjective feedbacks were obtained through observation and interview. Think-aloud Protocol.
Bateman et al. (34)	A user-centered design and analysis of an electrostatic haptic touchscreen system for students with visual impairments	2018	To detail the user-centered design and analysis of an electrostatic touchscreen system for displaying graph-based visual information to individuals who are visually impaired AND to present the usability study of the AD developed	For the UCD process the participants included technology experts with visual impairments, principals and teachers of a school for VIPS. For the usability study, 12 VIP	Interviews, preliminary tests, Usability test, Video analysis
Colley et al. (35)	Towards Inclusive External Communication of Autonomous Vehicles for Pedestrians with Vision Impairments	2020	To present an inclusive user-centered design for VPC, beneficial for both vision impaired and seeing pedestrians.	*N* = 6 participants (SD = 4.44; range: 45–56) years old, researchers conducted a between-subject study with *N* = 8 VIP and *N* = 25 seeing people.	Workshop, focus group. Testing: Time on street; • affective state: on a 7-point semantic scale using the self-assessment manikin (SAM), • cognitive load: raw NASA-TLX (36) on a 20-point scale, • subscale Predictability and Trust in Automation of the Trust in Automation questionnaire by Körber
Fidyka and Matamala (37)	Audio description in 360° videos Results from focus groups in Barcelona and Kraków	2018	To gather user feedback, through a series of focus groups, on how AudioDesciption (and secondarily AST) could be integrated in immersive content, both from the perspective of producers and consumers.	Barcelona: 6 participants: 2 end users (partially sighted), 3 audio describers and 1 technical expert. Kraków: 6 participants [3 end users (blind) and 3 audio describers]. Sex, age and other useŕs characteristics are reported.	Focus groups
Shi et al. (36)	Designing interactive 3D printed models with teachers of the visually impaired	2019	In this paper, we present two studies that investigate how to design I3Ms as effective teaching aids. In both studies	1st Workshop: 16 TVIs participated (1 with low vision, 2 blinds, 13 sighted). 2nd workshop: 19 TVIs (1 with low vision, 1 blind, 17 sighted). Age, experience and other participant´s data are reported.	Workshops, brainstorms, meettings. The remote meetings between the researcher and each TVI, and the TVIs feedback to their students’ were recorded. The data provided by the TVIs was the only one used because of “feasibility and privacy concerns”.
Doush and Pontelli (38)	Non-visual navigation of spreadsheets	2013	To measure the accuracy and the time needed when the user completes chart recognition tasks for bar, scatter, and line charts using the haptic/sound interface. T	7 unpaid students (3 females and 4 males) from New Mexico School for the Blind and Visually Impaired, 5 blind, 2 VI.	(1) Quantitative measurements(tima and aacuracy). (2) A questionnaire about each performed task and a questionnaire of the usability and accesibility of the system and potential suggestions to improve the system
Adebiyi et al. (39)	Assessment of feedback modalities for wearable visual aids in blind mobility	2017	The purpose of this paper is to report on a study comparing two types of ETA outputs (speech or tactile) in a group of blind test subjects.	All subjects were blind with regards to functional vision. Subject code, age, gender and visual diagnosis are shown. For 1st Experiment: 11 participants. 2nd experiment: 10 participants	A “person-in-loop” testing sessions. The variables measured were: Compliance (indoor and outdoor); Average Reaction Time, preferred walking speed with the cane (Control) and with each MFS (mobility feedback system). After all testing, SUS score.
Giraud et al. (40)	Web accessibility: Filtering redundant and irrelevant information improves website usability for blind users	2018	To test if this filtering provides a benefit in terms of cognitive load and usability according to the three usability criteria: effectiveness, efficiency and sat-isfaction	Participants were contacted via Internet through e-mail and forums dealing with disability. Fifty screen reader users voluntarily participated in these experiments.	The NASA-RTLX questionnaire measured cognitive load, three usability criteria were assessed (effectiveness, efficiency, and satisfaction), questionnaire “System Usability Scale”. Conducting semi-structured interviews would be necessary in order to collect the perceptions of users with blindness of such a tool
Lopes et al. (41)	MobiFree: A Set of Electronic Mobility Aids for the Blind	2012	To design a set of complementary electronic mobility aids for the blind, to cover as much as possible, his personal, near and far spaces: an improved long cane; the concept of a pair of sunglasses focus in the detection of head-level obstacles and a directional speaker to obtain echo information of surrounding elements.	Two blind people were invited	In order to give some feedback about the device and improvement tips, 1 person used the cane for more than two hours, and the other used the cane during a week. Both gave positive feedback and some functional tips.
Mascetti et al. (42)	Robust traffic lights detection on mobile devices for pedestrians with visual impairment	2015	This paper focuses on the problem of recognizing traffic lights with the aim of supporting a user with VIB in safely crossing a road.	The experiment involved 2 blind subjects and 2 low-visioned subjects (unable to see the traffic lights involved in the experiment).	A supervisor recorded whether the task was successfully completed and took note of any problem or delay in the process. The subjects were asked to answer a questionnaire.
Kammoun et al. (43)	Navigation and space perception assistance for the visually impaired: The NAVIG project	2012	The aim was to design and evaluate a powerful assistive device combining both micro- (sensing the immediate environment) and macronavigation (reaching a remote destination) functions.	21 VI users involved during all design steps. Selected using several criteria, including motivation to participate in the project, self-sufficiency in O&M, and some degree of practice with new technologies. Age: 16–65 years old; eight females and 13 males	The brainstorming sessions and discussions with VI users highlighted that an ideal system has to provide the best-suited level of audio guidance information.
Lee et al. (44)	From the Lab to People's Home: Lessons from Accessing Blind Participants’ Interactions *via* Smart Glasses in Remote Studies	2022	To explore ways to over come challenges associated with remote observations of blind participants’ interactions *via* video conferencing with smart glasses	12 blind participants, serving as a case study.	videos
Younis et al. (45)	A Smart Context-Aware Hazard Attention System to Help People with Peripheral Vision Loss	2019	This paper presents a new context-aware hybrid (indoor/outdoor) hazard classification assistive technology to help people with peripheral vision loss in their navigation using computer-enabled smart glasses equipped with a wide-angle camera.	5 visually impaired persons	3 Questionnaires: one about the challenges VIP face that would affect their QoL and their independent navigation; one about the hazardous situations while navigating. Dataset for hazard detection and classification; 1 Feedback regarding the sysem concept and alerts. Group meetting with patients.
Feiz et al. (46)	Towards Enabling Blind People to Fill Out Paper Forms with a Wearable Smartphone Assistant	2021	To present PaperPal, a wearable smartphone assistant which blind people can use to fill out paper forms independently.	For the WOZ pilot study: 8 blind participants. For the user study: 8 blind participants. Gender, age, skills, and other participantśdata were reported.	Semi-structured interview to gather demographic data, reading/writing habits, and prior experiences with assistive smartphone apps. The experimenter making notes throughout the video recorded session. A single ease question to each participant to rate the difficulty of assembling the holder and completing each form, on a scale of 1 to 7 open-ended discussion.

##### Quality assessment

Table 3 sums up the quality appraisal of the included studies following the MMAT criteria. Three papers were classified as qualitative, 15 as quantitative, and six as mixed methods studies. Only four papers met all quality criteria; nine papers met between 3 and 4 criteria, and 11 papers met between 1 and 2 quality criteria. When comparing the papers according to their type or nature, the ones that showed the highest compliance with the quality criteria were the qualitative studies. As for the quantitative studies, the first two criteria related to sampling strategy and representativeness of the sample were the least fulfilled. In this type of studies, in which probability samples would be expected, it was common to find undelineated information on the target population, lack of information on the selection criteria, non-probability samples and small sample sizes. On the other hand, as far as data analysis is concerned, the description of instruments such as questionnaires, their methods of analysis and the full presentation of their results are missing. In the mixed methods studies, the description of the application of the UCD approach took precedence over the definition of the mixed nature of the study. The methods of data analysis were clearly presented in the quantitative component of all the mixed methods studies, in the qualitative component only in one study.

**Table 3:** Quality assessment.

References	Qualitative studies					Quantitative descriptive studies					Mixed methods studies					Comments
	1.1	1.2	1.3	1.4	1.5	4.1	4.2	4.3	4.4	4.5	5.1	5.2	5.3	5.4	5.5	
Adebiyi et al. (39)	N/A	N/A	N/A	N/A	N/A	CT	N	Y	Y	Y	N/A	N/A	N/A	N/A	N/A	Sample strategy is not informed “Given the small sample size, an analysis of the within- subjects effect was computed using time to complete data to determine effect size.”
Aziz et al. (33)	Y	Y	CT	Y	Y	N/A	N/A	N/A	N/A	N/A	N/A	N/A	N/A	N/A	N/A	Inter-rater reliability is presented as the data analysis method. The data is analyzed reviewing themes, but it is not stated.
Bateman et al. (34)	N/A	N/A	N/A	N/A	N/A	N/A	N/A	N/A	N/A	N/A	Y	Y	CT	N	CT	The use of qualitative and quantitative methods is stated. The data analysis method of qualitative data is not specified.
Colley et al. (35)	N/A	N/A	N/A	N/A	N/A	N/A	N/A	N/A	N/A	N/A	Y	Y	Y	CT	Y	Although the study is not presented as a mixed methods study, the use of qualitative and quantitative methods is stated and described.
Connors et al. (32)	N/A	N/A	N/A	N/A	N/A	CT	N	Y	Y	Y	N/A	N/A	N/A	N/A	N/A	No information about sampling. “Relatively small” and as a “potential limitation particularly in terms of carrying out a correlation-based analysis”
Conradie et al. (21)	N/A	N/A	N/A	N/A	N/A	N/A	N/A	N/A	N/A	N/A	Y	Y	Y	N	CT	Authors fall short to explain the need of this approach. No detailed information about data analysis. Comparison of results between methods of different nature is not presented.
Doush and Pontelli (38)	N/A	N/A	N/A	N/A	N/A	CT	N	Y	CT	Y	N/A	N/A	N/A	N/A	N/A	No information about sampling strategy. Not specifics on target population.
Façanha et al. (22)	N/A	N/A	N/A	N/A	N/A	N/A	N/A	N/A	N/A	N/A	Y	Y	CT	CT	CT	Non- probabilistic sample. The method of analysis of qualitative data is not presented.
Feiz et al. (46)	N/A	N/A	N/A	N/A	N/A	CT	CT	Y	CT	Y	N/A	N/A	N/A	N/A	N/A	The study has small samples with no justification of sample size or sampling method. Quantitative variables and their methods of analysis are clearly explained. It is not very clear how the open-ended questionnaire is analysed in the initial part of the study. The feedback in the final part is estimated quantitatively.
Fidyka and Matamala (37)	Y	Y	Y	Y	Y	N/A	N/A	N/A	N/A	N/A	N/A	N/A	N/A	N/A	N/A	The paper presents sufficient information to verify compliance with the quality criteria according to the type of study.
Giraud et al. (40)	N/A	N/A	N/A	N/A	N/A	Y	Y	Y	CT	Y	N/A	N/A	N/A	N/A	N/A	The study meets the quality criteria according to its type. It should be noted that unlike other quantitative studies included, it does not collect feedback from users, but points out the need to conduct interviews in future studies to find out the perception of potential users.
Kammoun et al. (43)	N/A	N/A	N/A	N/A	N/A	CT	CT	Y	CT	Y	N/A	N/A	N/A	N/A	N/A	The paper presents a group of developments articulating results previously published separately. It does not go into details such as sampling strategies. Brain storming participatory design sessions are mentioned at the initial study phase. However, only quantitative data is presented.
Lee et al. (44)	N/A	N/A	N/A	N/A	N/A	Y	Y	Y	CT	Y	N/A	N/A	N/A	N/A	N/A	The paper presents sufficient information to verify compliance with the quality criteria according to the type of study.
Lopes et al. (41)	N/A	N/A	N/A	N/A	N/A	N	N	Y	CT	CT	N/A	N/A	N/A	N/A	N/A	No sampling strategy. Sample size (2). Superficial description for the data collection and analysis.
Mascetti et al. (42)	N/A	N/A	N/A	N/A	N/A	N	N	CT	CT	Y	N/A	N/A	N/A	N/A	N/A	No sample strategy. Sample size (4). Numerical measurements regarding to the devicès performance are analysed and presented. In contrast, the questionnaire applied after the tests is not described. Neither is its data analysis.
Mascetti et al. (30)	N/A	N/A	N/A	N/A	N/A	CT	CT	Y	N	Y	N/A	N/A	N/A	N/A	N/A	Non-probability sampling, No description of the target population. The use of qualitative methods is reported. However, they turn out to be Likert-scale surveys, to which a frequency analysis is applied.
Mattheiss et al. (24)	N/A	N/A	N/A	N/A	N/A	N/A	N/A	N/A	N/A	N/A	Y	Y	Y	Y	Y	The paper presents sufficient information to verify compliance with the quality criteria according to the type of study.
Najjar et al. (31)	N/A	N/A	N/A	N/A	N/A	CT	N	Y	Y	Y	N/A	N/A	N/A	N/A	N/A	No information about sampling strategy. Not specifics on target population.
Nimmolrat et al. (26)	N/A	N/A	N/A	N/A	N/A	Y	Y	Y	Y	Y	N/A	N/A	N/A	N/A	N/A	The study stands out for its clarity in presenting the sampling technique and a sample size in line with its aim. It applies open-ended questionnaires through interview techniques, but analyses the data according to frequencies.
Ntakolia et al. (23)	N/A	N/A	N/A	N/A	N/A	N/A	N/A	N/A	N/A	N/A	Y	Y	CT	N	CT	Qualitative and quantitative methods are applied for the identification of user needs. However, priority is given to UCD approach implementation, not to the mixed nature of the study. The qualitative component is presented superficially, showing issues addressed and results but without explaining their methods of analysis. The usability test does not include VI users, without giving reasons.
Sánchez (25)	N/A	N/A	N/A	N/A	N/A	CT	CT	Y	CT	Y	N/A	N/A	N/A	N/A	N/A	Non-probabilistic sampling. No information about the target population. Small sample.
Shi et al. (36)	Y	Y	Y	Y	Y	N/A	N/A	N/A	N/A	N/A	N/A	N/A	N/A	N/A	N/A	The paper presents sufficient information to verify compliance with the quality criteria according to the type of study.
Yeh and Yang (27)	N/A	N/A	N/A	N/A	N/A	Y	N	Y	Y	Y	N/A	N/A	N/A	N/A	N/A	Small non-probabilistic sample. Reasons presented in the limitations section
Younis et al. (45)	N/A	N/A	N/A	N/A	N/A	CT	CT	Y	CT	Y	N/A	N/A	N/A	N/A	N/A	The study analyses series of database. The subjects participation is in an exploratory study.

MMAT items: 1.1. Is the qualitative approach appropriate to answer the research question?; 1.2. Are the qualitative data collection methods adequate to address the research question?; 1.3. Are the findings adequately derived from the data?; 1.4. Is the interpretation of results sufficiently substantiated by data?; 1.5. Is there coherence between qualitative data sources, collection, analysis and interpretation?; 4.2. Is the sample representative of the target population? 4.3. Are the measurements appropriate?; 4.4. Is the risk of nonresponse bias low?; 4.5. Is the statistical analysis appropriate to answer the research question? 5.1. Is there an adequate rationale for using a mixed methods design to address the research question?; 5.2. Are the different components of the study effectively integrated to answer the research question?; 5.3. Are the outputs of the integration of qualitative and quantitative components adequately interpreted?; 5.4. Are divergences and inconsistencies between quantitative and qualitative results adequately addressed?; 5.5. Do the different components of the study adhere to the quality criteria of each tradition of the methods involved?.

Values: Y: yes; N: no; CT: Cańt tell N/A.

### Adoption of UCD principles

The aim of this systematic review is to examine and describe the application (or absence of application) of the ISO 9241-210’s principles in AT developments for persons with VI, based on searches in multidisciplinary databases. Here, we present an in-depth analysis of findings pertaining to the development and adoption of the UCD principles.

The available evidence shows that papers documenting the development of AT for persons with VI tend to present a more detailed description of the state of the art in terms of the systems requirements than a proper characterization of the context of use or preferences and needs of the target user. Also, there is a strong emphasis on usability-oriented studies (64.29%). AT developments tend to engage users mostly at the end of the process to test if the product can be used. More than one third (35.71%) of the articles did exactly that. While 21.4% presented usability evaluation and results as part of the user-centred design process. Another 21.4% aimed to apply the user-centred design process in the early stages of the design or development process, looking at the feasibility of using the device and highlighting the compatibility and advantages of using participatory design methodologies with the UCD approach.

Table 4 succinctly outlines the results of the application of the principles of ISO 9241-210 in the papers included in this review. The following sections expand on the findings of the detailed peer review on the application of this standard, grouping them under each of the six principles.

**Table 4:** Data extraction from reviewed papers on the application of the principles of ISO 9241-210 in assistive technology design or development processes.

References	Principles of the ISO 9241-210					
	5.2 The design is based upon an explicit understanding of users, tasks and enviroments	5.3 Users are involved throughout design and development	5.4 The design is driven and refined by user-centred evaluation	5.5 The process is iterative	5.6 The design addresses the whole user experience	5.7 The design team includes multidisciplinary skills and perspectives
Conradie et al. (21)	Yes	Yes	Yes	Yes	Yes	Not described
Façanha et al. (22)	Yes	Yes	Yes	Iteration is described in the theoretical framework, not in the development process	Yes	This group had four computing undergraduate students, an assistive technology researcher and an ophthalmologist.
Ntakolia et al. (23)	Yes	Users are involved in design and development, not in evaluation	Yes, but usability test was made with blinded participants	Yes	Yes	Not described
Mattheiss et al. (24)	Yes	Yes	Yes	Yes	Yes	The design team is not described.
Sánchez (25)	Yes	Yes	Yes	Yes	Yes	The research team is not descibed, Authors described abundant team experience regarding interface design for blind children was also used in this design process.
Nimmolrat et al. (26)	Yes	Yes	Yes	No, but suggestions are made for improvements	Yes	Not described
Yeh and Yang (27)	Yes	Yes	Yes	Yes	No	Not described
Mascetti et al. (30)	No	Users involved in preliminary and usability tests	No	Yes	No	The design team is not described.
Najjar et al. (31)	No	Users are involved in usability	No	Yes	No	Not mentioned
Connors et al. (32)	Yes	Yes	Yes	Not described	Not described	Not described
Aziz et al. (33)	No	Users are involved since the usability test of the prototype	No	The intention is declared by the authors.	Yes	Not described
Bateman et al. (34)	Yes	Yes	Yes	Yes	Yes	The design team is not described.
Colley et al. (35)	Yes	Yes	Yes	Iteration is described in the theoretical framework, not in the development process	Yes	Not mentioned
Fidyka and Matamala (37)	Yes	Yes	Yes	Not declared	Yes	Not described
Shi et al. (36)	Yes	Yes	Yes	Yes	Yes	One of the researchers is an expert in education for students with VI. An accessibility specialist was included in the researchers team to assess the usability of the application before delivering it to the TVIs.
Doush and Pontelli (38)	No	Involved in the usability test	No	Yes	No	Not described
Adebiyi et al. (39)	No	Users are involved in tests to compare audio and vibotactile ETA outputs.	No	Intention declared	No	Not described
Giraud et al. (40)	No	Involved in the usability test	Yes	Yes	No	Not described
Lopes et al. (41)	No	In the paper only is stated user participation in the evaluation	No	Yes	No	Collaboration with the Department of Communication and Arts of the Aveiro University, In the design process, is reported.
Mascetti et al. (42)	No	Users are involved in usability tests	No	Yes	No	The design team is not described.
Kammoun et al. (43)	Yes	Yes	Yes	Yes	Yes	NAVIG brings together two research laboratories in computer science and information technology and one research laboratory in human perception. IRIT-Elipse, the project leader, is an interdisciplinary research group in Human Computer Interaction (HCI).
Lee et al. (44)	Yes	Yes	Yes	Yes	No	Our team, including four sighted researchers and one blind researcher.
Younis et al. (45)	Yes	Yes	Yes	Intention declared	Yes	Authors declared research collaboration with the Department of Health Services Research
Feiz et al. (46)	Yes	Yes	Yes	Yes	Yes	Not described

### The design is based upon an explicit understanding of users, tasks, and environments

In reviewing the application of this principle in the available evidence, we sought compliance with the following points: identification of user and stakeholder groups, understanding of users’ needs and description of the context of use: “specified users, having specified goals, performing specified tasks”.

All the reviewed articles reported the participation of VI users, even though six studies complemented their samples with non-VI participants. Regarding sample sizes, the number of participants varies from two to 128 (55 VI, 71 non-VI), being 12 participants the number that appears the most often. Only seven articles reported sample sizes with more than 12 users. There is no clear rationale for why and how these samples were designed and selected, especially when considering the quantitative methodologies. Only one paper, Nimmolrat et al. reported the sampling strategy and inclusion criteria for a sample of 60 participants (26).

Quantitative standards regarding sampling were not observed either. Mascetti et al, reported difficulties in recruiting test subjects with VI or blindness (30). Under that argument, the paper added non-VI participants to the study and reported on results that merge data from both non-VI individuals and VI individuals. Najjar et al., whose sample consisted of 10 non-VI and seven VI participants, noted their limitations without being specific or addressing bias on the data analysis (31). Connors et al. acknowledged that their sample size (7 blind adolescents) was “relatively small” and limited to carry out a correlation-based analysis (32).

When qualitative methods were applied, no standard sampling techniques nor quality assurance practices for qualitative sampling were reported, e.g., characterization of patterns, and variations among the participants, data saturation. Studies such as Aziz et al., argued that the sample size (8) was sufficient considering its qualitative nature, without providing any further rationale (33). Furthermore, Conradie et al. claimed that two focus groups (sample sizes 9 and 12) “served to reveal the experiences and knowledge of blind persons” to the researchers which make it possible to sketch broad user needs within the target group and specifying varying degrees of mobility needs and assistive device demands (21).

In terms of characterising users, participants' details were poorly described. The sex of the sample members was reported by 19 papers, the same number of articles stated the age of the participants, four of the studies were addressed to minors (8–17); two other papers reported participation of teenagers and young adults; the rest of the studies included adults only in different age ranges (18–78). Other types of data reported were: the participant's skills related to the use of the designed AT (14 papers); the education level of the participants (10 papers), their occupation (five papers), the number of years lived with visual disability or the year when the disability was acquired (five papers), and the use of aids (two papers). Environment of use was often not mentioned. Only one paper reported an analysis of the physical environment in which the product will be used, the user's social and organisational milieu and the technical environment and associated technical constraints (23).

As for stakeholder identification, 8 papers mentioned the involvement of stakeholders. Four papers declared the participation of academics (principals, teachers and O&M trainers), three studies included representatives from disabled people's organisations, and four studies included technology experts (22–25, 34–37). Bateman et al., included all the above (34).

### Users are involved throughout the design and development

All of the reviewed articles reported user involvement, but rarely throughout all of the stages of the design process. Four of the studies present data from the design phases in which participants were actively involved (21, 24, 35, 37). Their involvement included “in-depth requirements analysis” of users and stakeholders' feedback through a series of UCD comprehensive methods. Mattheiss, et al. first centred on analysing the requirements in the areas of Orientation and Mobility (O&M) training and accessible video game play to later work on the first iterations of the design, implementation, and evaluation of the developed game editor (24). In this case, authors declared the involvement of children (end-users) as design partners.

User participation in the final stages, namely for evaluating the solutions, was stated by 5 studies: 4 in usability testing (33, 38–40) and 1 in field testing (41). Involvement, both in the design and testing phases, was reported by 14 studies (22, 25–27, 30–32, 34, 36, 42–46). Although, it is pertinent to point out that: on the one hand, some studies mention the involvement of the participants at the beginning and at the end of the process, but not in all the stages of the process. On the other hand, how users were involved tend to be unclear and reporting of such involvement tends to be rather superficial, for example, Najjar et al. mentioned the identification of potential users' requirements, but these are not presented in the paper (31), instead a previous study is referred to. Nimmolrat et al. provide a better description of users' participation during the design process (26). Ntakolia et al. detailed user's participation in the design and development phases, however, usability testing was done with blindfolded non-VI participants only (23).

### The design is driven and refined by user-centred evaluation

The use of user-centred evaluation tends to be more explicit, explained and applied in studies that used qualitative methods, such as behavioural observation, think aloud techniques, in-depth interviews, and focus groups, among others. These kinds of evaluation methods allow the user's perspective to be addressed early (21–26, 33, 34, 37, 46). The analysis of the context of use could determine the user's needs against which the preliminary design solutions will be tested.

Usability evaluations reported on the evidence collected included both quantitative and qualitative methods. There is a stronger emphasis on quantitative scales to assess usability, such as the System Usability Scale. In addition, usability was assessed in terms of the performance of the technology and other quantitative variables related to efficiency (time) and effectiveness. Except for the studies by Najjar et al. and Giraud et.al, the studies which applied quantitative methods for usability testing, also reported userś feedback without specifying the methods used to gather that data (31, 40), e.g., Lopes et al., stated that subjects had the chance to use the device and were asked to give feedback, but did not describe the methods for data collection (41).

There is a lack of real-world scenarios when evaluating AT. Some studies claim that this was because they are focused on preliminary solutions, and in some others, because the study has pure research and non-commercial orientation (24, 27, 31, 40). Ntakolia et al., excluded users from its evaluation process of the prototype, reporting that future research would include VI users for the usability test (23).

### The process is iterative

In reviewing compliance with iteration, which dictates the iterative repetition of a sequence of steps until the desired outcome is achieved, it is important to remember that not all the included articles report the complete UCD process, but some focus on design and several, as presented initially, are limited to the usability evaluation of prototypes. Thus, all the studies reported iterations, or the intention to make them, based on the feedback gathered from studies' participants. Iteration involves not only the prototype but also the descriptions and specifications, the refinement of information from the feedback obtained during the development process and in usability testing, is also considered. From this perspective, a noteworthy study on this subject is that of Bateman et al. where design and re-designs were submitted to preliminary tests with expert users (34). Finally, the usability test conducted with 12 students confirmed that the previously expressed needs regarding accessibility and effectiveness were met. The authors went beyond mentioning that an iterative UCD process was carried out, in fact, they went on to explain the information gathered and the stakeholder's characteristics through every round of interviews. The iterations made and the preliminary test results were also detailed. In other words, iterations were placed in the context of use.

Another interesting example is the study by Shi et al., where two studies were conducted to understand how to design effective, interactive 3D models for education purposes for blind students (36). In the first study, two design workshops were performed with teachers of VI students (TVIs) in which suggestions from conceptual designs were aggregated. Then, the second study was performed with three teachers of VI students, not only to design, but to deploy sample interactive 3D models over seven weeks. In-depth work with individual TVIs, and deployment of interactive 3D models in their classrooms were reported by the researchers, resulting in improvements to the prior system and mobile application development that supports the use of interactive 3D printed models in an educational setting. Additionally, the authors stated that based on the feedback from the second study, the mobile application could be further improved.

Although in less depth than the cases previously discussed, Conradie et al. and Mattheiss et al. highlighted the importance of rapid prototyping in the execution of iterations (21, 24). Adebiyi et al. and Feiz et al. emphasised the effectiveness of the “Wizard of Oz” technique in achieving development improvements (39, 46).This technique consists of a tactic used for low fidelity prototyping in which the participant receives instructions in order to perform tasks while testing a prototype, and a human simulates the behaviour of the completed AT. For example, for a navigation device, a person will simulate the task that the device will perform by providing vocal instructions to the users.

In turn, Doush and Pontelli reported “iterative modifications have been applied to the system based on empirical studies carried out with the participation of sighted and blind users”. However, they do not describe the iterations performed or how these studies were conducted, nor do they explain why non-visually impaired participants were involved (38). Likewise, Mascetti et. al, stated that “during the design of the auditory guiding modes several test subjects were asked to use the application and provide feedback” this was done via informal test (30).

### The design addresses the whole user experience

ISO 9241 stresses that usability goes beyond “making products easy to use”, by considering perceptual and emotional aspects as keys to understanding the user's experience from their own perspective.

Still, several studies assessed usability mainly by considering parameters such as ease of use, accessibility, or satisfaction with the device (30, 31, 38–40, 42). These studies applied quantitative scales. To have information to improve the device, three papers reported to have included questionnaires or open-ended questions (not described in the papers) (30, 38, 42). In yet another case, in which only System Usability Scale (SUS) was applied, feedback from users was reported as results of “anecdotal comments” (39).

Mascetti et al., reported as a result of feedback from participants after evaluation of the prototype, that they did not desire to hold a mobile phone in one hand while holding a cane in the other (42). This type of information evidences that the characterisation of users' needs and preferences was not carried out at an early stage and therefore, users' previous experiences and perspective were not addressed.

Other feedback refers to the time the user needs to get familiar with the device, the need for more training time was expressed by the participants in the studies conducted by Doush and Pontelli and by Mascetti et al. (38, 42, 46). It was also stated by Najjar et al. (31). On the other hand, although Giraud et al., did not include feedback within the methods or results, they did express the future need of conducting semi-structured interviews “in order to collect the perceptions of users with blindness of such a tool (advantages, risks, opportunities)” (40).

Alternatively, preferences and expectations were mainly assessed in the studies of Mattheiss et al., and Aziz et al.: design for skills development in VI children through an interactive learning material and a videogame, respectively (24, 33).

Furthermore, Sánchez obtained feedback from users regarding their emotions (25). Colley et al., also considered affective state variables, namely “control over the situation” in the analysis (35). Nimmolrat et al., assessed satisfaction with the functionality of the application through interviews (26). Finally, eight studies (28.57%) based their development on the available literature and did not include collecting any empirical data.

### The design team includes multidisciplinary skills and perspectives

The large majority of studies did not report multidisciplinary skills and perspectives. Only two studies described the research team. Facanha et al., stated that the design team included four undergraduate students in the computer sciences, an assistive technology researcher and an ophthalmologist (22). Shi et al., mentioned that one of the researchers of the team is an expert in education for students with visual impairments, and that they included an accessibility specialist (36). Nevertheless, some authors did report collaborations: Kammoun et al., declared the participation of different engineering research groups in human perception, human-computer interaction, audio and acoustic, and spatial cognition and perception. Further, the authors mentioned the project leader, is an interdisciplinary research group in Human Computer Interaction (43). Other reports of collaborations outside the engineering team are: Lopes et al., who mentioned a collaboration with the Department of Communication and Arts of Aveiro University (41) and Younis et al., who declared a research collaboration with the Department of Health Services Research in the UK (45).

## Discussion

The literature reports a growing trend in the application of user-centred design in the development of assistive technology for the visually impaired persons (47). However, the results show that evidence on the effective implementation of UCD with VI on the design of AT is scarce. Publications show that the principles of the ISO 9241-210 (user-centred design) tended to be not fully applied, despite being called guiding principles and despite the increasing availability of models and frameworks that could facilitate their application (48).

### The focus was on the system requirements, not its user

The information on the system architecture reported in the state of the art in the analysed studies, was prioritised over the participants' needs with respect to AT. For these papers, it was common not to find specifics on sample size calculation and participant's selection. Participation of potential users was low and was accompanied by a superficial description of their profiles. Users' involvement was reported mainly in usability assessment at the end of the process rather than in design phases, and when users’ involvement was reported in design phases, it was usually not thoroughly described. This contrasts with the extent in which technological aspects of the development were informed. Also, a stronger focus on the verification of the system, over its validation, was observed. According to quality management standards, such as the ISO 9001, independent validation and verification (V&V) processes need to be performed to determine if a developed system meets the defined requirements and specifications and fulfils its intended purpose (49, 50). Specifically, the verification process focuses on the system's requirements (“Did we developed the system right?”) while the validation process focuses on the system's worthiness, i.e., if it fulfils its intended purpose, user expectations, etc. (“Did we developed the right system?”) (51).

For usability assessment, most studies used surveys and quantitative methods to gather information. Though standard parameters of quality on those methods, such as rationale for power and limitation of the sample size calculation, were not met. Feedback from users, when present, took the form of “informal”, “casual” or “anecdotal” data. Moreover, in these studies iterations are often mentioned in the evaluation phase and not in the design phase. At this point, it is important to emphasise that according to the ISO 9241-210, iterations should be done throughout the process and not only at the evaluation stage.

Regarding usability, ISO 9241-210 states the need to go beyond the concepts of ease of use and effectiveness, and to incorporate userś experience. In this perspective, the standard recommends to consider the userś skills, habits and personal goals, as well as emotional aspects and experiences of previous solutions or alternatives. Notwithstanding, the studies under consideration fall short in assessing the whole users' experience, there was little or no information on the social and environmental context in which these devices were intended to be used. In this regard infrastructural constraints such as internet availability, road safety or social aspects like stigma are not accounted for.

### Moving towards better understanding of the final users

Characterization of user needs was often unstructured, lacked robustness or tended to be underreported. This trend has been previously observed in the study of requirements elicitation techniques (48). This was also observed in the present review. Among the reasons given to justify such behaviour are limited resources, time and endeavour to conduct a thorough requirements assessment process (48). Similarly, some of the studies in this review reported major logistical challenges in recruiting participants.

There is a growing number of articles that seek to better engage with users of AT. This was generally achieved either because they took care to obtain larger samples under previously defined selection criteria or because they selected more appropriate methods (qualitative or mixed) with respect to the objective pursued, or due to both reasons (23–26, 43). It is also pertinent to highlight the importance of having included stakeholders in these studies (22–26, 36, 37, 43, 44).

Researches did not fully apply all ISO 9241-210's principles. However, it can be argued that a better compliance to the first principle (*the design is based upon an explicit understanding of users, tasks, and environments*) increased the probability of applying the subsequent four principles. The fact that some studies integrated participatory design approaches into the methodology boosted the involvement of participants in the whole process (22, 24, 26, 27, 36, 44). The participation of both potential users and stakeholders in the early design phases and throughout the process, as well as the type of instruments applied to collect information, allowed the design to be “driven and refined by user-centred evaluation”. Iterations were reported both in the information collected to guide the design, and in the prototypes.

Regarding usability, in addition to the application of validated surveys and the analysis of system performance parameters, qualitative methods were used to obtain feedback from users in a more systematic and deeper way, and to compare it with the initial information from the context of use. In some cases, the user experience was assessed in a more comprehensive way by considering the emotions evoked through interaction during the prototypes assessment (24, 33, 35).

### Multidisciplinary skills and perspectives

So far, the application of five of the six principles of the ISO 9241-210 in the reviewed articles has been discussed. Regarding the application of the last principle “The design team includes multidisciplinary skills and perspectives” in the reviewed studies, this is where the least evidence was reported. Although the standard does not define the need for broad heterogeneity of the disciplines involved in the process, since it is designed to guide processes of different natures, it is understood that disciplines from diverse fields are needed to elicit and comprehend userś needs and to address the system's requirements. Multidisciplinary teams would not only allow dealing with the issues related to technology but also those that have to do with the users' functionality, and above all, it would facilitate the mixed methodological approach.

As for the literature, in addition to designers and engineers, it is proposed in specific cases to work with clinicians, health professionals or rehabilitation professionals and with Commercial specialists (47, 49).

### From user centred to person centred

Literature addresses two streams of user-centred design, one in which the “user” is placed at the centre of the design process and another, which focuses on the “person” (52). The main difference lies in the fact that the first considers the interaction between the user and the product and “is concerned with ensuring that artifacts function as intended by the designers”. While the latter also accounts for context-determined interactions and focuses on “enabling many individual or cultural conceptions to unfold into uninterrupted interfaces with technology.” Giacomin et al., add that products acquire meaning when used by persons, and that it is the understanding of that meaning that should guide the design. In their words, “the natural focus of questions, insights and activities is on the people for whom the product, system or service is intended, rather than on the designer's personal creative process or on the material and technological substrates of the artefact” (52). We might say that most articles followed the first trend (user-centred), while there is less evidence of the second understanding (person-centred) when it comes to AT.

## Limitations

Although this systematic review process was based on best practice in conducting systematic reviews (PRISMA), like any research, has its limitations. Some studies on the topic may have been missed, either because they were not found within the four selected databases or because there is a chance that some studies are not covered by the search string.

To reduce this risk, we selected multidisciplinary electronic databases relevant to the topics addressed. The key terms and search string were tested. In addition, as reported in the methodology, strategies to increase inter-rater reliability in the studies selection and data extraction were carried out.

## Conclusion

This review explores how well the principles of ISO 9241-210 are applied in the case of AT. As for the implications, on the one hand, it highlights that the application of the UCD approach is not standardised in the field of AT design for the visually impaired. Although there is a standard that guides the implementation of the approach and has been thoroughly reviewed by ISO experts, it has not been embraced in this field. On the other hand, there is also a lack of methodological rigour in understanding the needs of users in their context, revealing that people are not at the centre of the process in a generalised manner.

These findings confirm the outcomes of the studies quality appraisal. The areas of concern in the quality of the studies include non-probabilistic and small samples in the quantitative studies. Further, lack of rigour in the analysis and description of some questionnaires and in the collection of feedback from users. Absence of description of the methodology of mixed methods studies and omission of the analysis of qualitative data and methods. These results serve as an input to understand the nature of the problem and to look for solutions that aim to improve the research processes and therefore the development of better products.

Furthermore, it is evident that the developments are carried out in a disarticulated manner, so that recommendations made by international authorities on the subject, such as those given by WHO in GREAT (7), are also disregarded.

Based on these findings, we emphasise the need to pay greater attention to the principle: “Users are involved throughout the design and development”, meaningfully engaging with users would lead to better identification of their needs and preferences. It shall also improve the possibility to have better recruitment procedures, representative samples and more representative and robust results.

Engaging with users will require a broad level of expertise and full implementation of ISO 9241-210 principle 5.7 “The design team includes multidisciplinary skills and perspectives”. Transdisciplinarity could have reduced methodological flaws observed in the literature today. Transdisciplinarity shall open the possibility of cross-fertilization between the different fields of knowledge and in conjunction with people with visual impairments as potential direct users and with their stakeholders. The application of this principle shall enable design teams to include not only diverse classes of engineers, but also designers, health and rehabilitation professionals, social scientists including disability scholars, anthropologists, and economists, among others. Such teams shall be better equipped to develop and apply a range of methodologies that understand the social, physiological, cultural and technological needs of the target users and develop AT that responds to them. The strengthening of this last principle of the standard would lead the work towards the consolidation of adequate methodologies to gain a better understanding of how AT could enable visually impaired people to live the lives they would like to live.

Design of AT should be focusing on enhancing the user's agency, bodily integrity, and capabilities, and not trying to “fix disabled bodies”. Evidence collected suggests that assistive technology has focused on functional deficiency solely, namely impairment rather than in enhancing wellbeing for its users (6, 53). The latter seems to be the prevalent approach today as users are for the most part not meaningfully included in the design and development and only call to test a final product that aims to provide a “solution”. “Nothing About Us Without Us” should resonate with the design, development and implementation of any technological development that concerns persons with disabilities.

## Data Availability

The original contributions presented in the study are included in the article/Supplementary Material, further inquiries can be directed to the corresponding author.
