# Supplementary material for: Assessing the implementation of user-centred design standards on assistive technology for persons with visual impairments: a systematic review
Source: Front Rehabil Sci. 2023 Sep 6;4:1238158. doi: 10.3389/fresc.2023.1238158 (PMC10511648; doi:10.3389/fresc.2023.1238158)
Supplement: Supplementary file 1 [file Datasheet1.pdf]

Assessing the implementation of user-centred design standards on assistive technology for persons with visual impairments: a systematic review

To enable PROSPERO to focus on COVID-19 submissions, this registration record has undergone basic automated checks for eligibility and is published exactly as submitted. PROSPERO has never provided peer review, and usual checking by the PROSPERO team does not endorse content. Therefore, automatically published records should be treated as any other PROSPERO registration. Further detail is provided here.

Citation

Luisa Maria Ortiz-Escobar, Mario Chavarria, Samia Hurst-Majno, Michael Ashley Stein, Anthony Mugeere, Klaus Schönenberger, Minerva Rivas Velarde. Assessing the implementation of user-centred design standards on assistive technology for persons with visual impairments: a systematic review. PROSPERO 2022 CRD42022307466 Available from: https://www.crd.york.ac.uk/prospero/display_record.php?ID=CRD42022307466

Review question

Do user-centered designed assistive technology developments for persons with visual impairments apply human-centred design principles of the ISO 9241-210?

Searches

For this review, the following databases will be used: Web of Science, PubMed, ScienceDirect, and Scopus As search terms will be used: Visual impairments (blindness and low vision), user-centered design, assistive technology.

The search period will be between January 2012 and April 2022. Publications written in English, Spanish, Portuguese, and French will be included.

Types of study to be included

Inclusion criteria:

• Topic of study: papers describing the design and/or development process of user-centered designed assistive technology for visually impaired persons.

• Type of scientific material to analyze: Peer-reviewed journals. Any type of research design: experimental, descriptive, or analytic research design. Except for letters, editorials, and non-empirical studies. It will include systematic reviews and meta-analysis.

• Studies available in English / Spanish/ Portuguese / French

• Full text available

• Full conference papers

• Sufficient information on early design/development

Exclusion criteria:

• Articles that are not mainly addressed to persons with visual impairments

• Articles describing assistive technology design or developments addressed for persons with visual impairments without any consideration to the user-centered design approach

Condition or domain being studied

Assistive technology developments for persons with visual impairments (blindness or low vision).

Assistive technology, as defined by WHO’s Global Cooperation on Assistive Technology (GATE), “is the application of organized knowledge and skills related to assistive products, including systems and services. Assistive technology is a subset of health technology.”

Participants/population

Persons with visual impairments (blindness or low vision)

Intervention(s), exposure(s)

User-centered designed assistive technology developments for persons with visual impairments.

Comparator(s)/control

Not applicable

Context

We aim to include studies within all contexts and settings, involving user-centered designed assistive technology developments for persons with visual impairments in daily life activities whether in indoor or outdoor settings and for any purposes (i.e. academic, occupational, recreational, among others), in low, middle or high-income countries.

Main outcome(s)

Description of the application (or absence of application) of the ISO 9241-2190´s principles in assistive technology developments for persons with visual impairments

• Understanding of users, tasks and enviroments: methods, instruments and tools to identify user´s requirements

• Users involvement in the design process: Phases in which end-users were included and their kind of participation

• Design driven by user-centered evaluation

• Iteration processes: number of iterations (if applicable)

• User experience: methods, instruments and tools to assess user experience

• Disciplines involved in the development process

Measures of effect Not applicable

Measures of effect

Not applicable

Additional outcome(s)

Identification of methodological tools for the data collection of user´s needs and experiences in the development of user-centered designed assistive technology developments for persons with visual impairments

Measures of effect

Not applicable

Data extraction (selection and coding)

The following data will be extracted:

• Data about the publication (authors, title of the article and the journal), aims, methods, design approaches (usability testing, workshops, interviews, focus groups, think-aloud, observation, etc.), frameworks, and studies design.

• Data about participants: sample size, socio-demographic characteristics, inclusion and exclusion criteria, type of impairments (low vision or blindness).

• Setting: country

• And the application of ISO 9241-210´s principles in assistive technology developments.

All search results will be imported into an EndNote database. Duplicates will be removed. Abstracts and titles that are noticeably unrelated to the review topic will be dismissed. Two researchers will screen independently the titles and abstracts against the eligibility criteria and select those that meet the inclusion criteria. Full-text reports will be retrieved and again, assessed for final eligibility. Reasons for excluding full-text reports will be documented. The selected studies will be analyzed with a standardized data extraction form. Any disagreement during the selection process will be discussed in a consensus meeting, with a third reviewer who will help to help to solve the discrepancies.

Risk of bias (quality) assessment

An adapted checklist will be applied independently by two researchers to perform risk of bias assessment; any disagreement will be resolved through discussion with a third researcher.

Strategy for data synthesis

All search results and their respective reasons for inclusion or exclusion will be documented through The PRISMA flowchart. The body of evidence will be analyzed qualitatively by the themes stated in the “data extraction” regarding the ISO 9241-210´s principles application and will be presented through a descriptive overview. The quantitative data (publication data, data about the participants, setting, and principles application) will be presented through descriptive statistics tools, such as frequency tables and others, if needed, according to the volume of evidence resulting from the selection process.

Analysis of subgroups or subsets

None

Contact details for further information

Luisa Maria Ortiz-Escobar

lmortiz@uao.edu.co

Organisational affiliation of the review

Universidad Autonoma de Occidente

https://www.uao.edu.co/

Review team members and their organisational affiliations

Luisa Maria Ortiz-Escobar. Universidad Autonoma de Occidente

Mario Chavarria. EssentialTech Centre of Ecole Polytechnique Fédérale de Lausanne EPFL

Samia Hurst-Majno. University of Geneva

Michael Ashley Stein. Harvard University

Anthony Mugeere. Makerere University

Klaus Schönenberger. EssentialTech Centre of Ecole Polytechnique Fédérale de Lausanne EPFL

Minerva Rivas Velarde. University of Geneva

Type and method of review

Systematic review

Anticipated or actual start date

30 November 2021

Anticipated completion date

31 October 2022

Funding sources/sponsors

Fondation Gelbert

Grant number(s)

State the funder, grant or award number and the date of award

0751-2020

Conflicts of interest

Language

English

Country

Colombia, Switzerland

Stage of review

Review Ongoing

Subject index terms status

Subject indexing assigned by CRD

Subject index terms

Humans; Self-Help Devices; Vision Disorders

Date of registration in PROSPERO

10 September 2022

Date of first submission

30 August 2022

Stage of review at time of this submission

Stage	Started	Completed
Preliminary searches	Yes	No
Piloting of the study selection process	Yes	No
Formal screening of search results against eligibility criteria	Yes	No
Data extraction	No	No
Risk of bias (quality) assessment	No	No
Data analysis	No	No

The record owner confirms that the information they have supplied for this submission is accurate and complete and they understand that deliberate provision of inaccurate information or omission of data may be construed as scientific misconduct.

The record owner confirms that they will update the status of the review when it is completed and will add publication details in due course.

Versions

10 September 2022

10 September 2022
